# Enhancing *Solanum lycopersicum* Resilience: Bacterial Cellulose Alleviates Low Irrigation Stress and Boosts Nutrient Uptake

**DOI:** 10.3390/plants13152158

**Published:** 2024-08-04

**Authors:** Noelia De la Cruz Gómez, César Poza-Carrión, Lucía Del Castillo-González, Ángel Isidro Martínez Sánchez, Ana Moliner, Inmaculada Aranaz, Marta Berrocal-Lobo

**Affiliations:** 1 Centro para la Biodiversidad y Desarrollo Sostenible (CBDS), Universidad Politécnica de Madrid, 28040 Madrid, Spain; n.delacruz@alumnos.upm.es (N.D.l.C.G.); cpoza@cnb.csic.es (C.P.-C.); l.delcastillo@alumnos.upm.es (L.D.C.-G.); aims@alumnos.upm.es (Á.I.M.S.); 2 Arquimea Agrotech S.L.U, 28400 Madrid, Spain; 3 Escuela Técnica Superior de Ingeniería Agronómica, Alimentaria y de Biosistemas, Universidad Politécnica de Madrid, 28040 Madrid, Spain; ana.moliner@upm.es; 4 Instituto Pluridisciplinar, Departamento de Química en Ciencias Farmacéuticas, Universidad Complutense, 28040 Madrid, Spain; iaranaz@ucm.es

**Keywords:** bacterial cellulose, *Solanum lycopersicum*, biopolymer, drought, bioestimulant, plant nutrient uptake, water use efficiency, trancriptomics, water-holding capacity, tomato

## Abstract

The use of natural-origin biomaterials in bioengineering has led to innovative approaches in agroforestry. Bacterial cellulose (BC), sharing the same chemical formula as plant-origin cellulose (PC), exhibits significantly different biochemical properties, including a high degree of crystallinity and superior water retention capacity. Previous research showed that natural-origin glucose-based chitin enhanced plant growth in both herbaceous and non-herbaceous plants. In this study, we produced BC in the laboratory and investigated its effects on the substrate and on *Solanum lycopersicum* seedlings. Soil amended with BC increased root growth compared with untreated seedlings. Additionally, under limited irrigation conditions, BC increased global developmental parameters including fresh and dry weight, as well as total carbon and nitrogen content. Under non-irrigation conditions, BC contributed substantially to plant survival. RNA sequencing (Illumina^®^) on BC-treated seedlings revealed that BC, despite its bacterial origin, did not stress the plants, confirming its innocuous nature, and it lightly induced genes related to root development and cell division as well as inhibition of stress responses and defense. The presence of BC in the organic substrate increased soil availability of phosphorus (P), iron (Fe), and potassium (K), correlating with enhanced nutrient uptake in plants. Our results demonstrate the potential of BC for improving soil nutrient availability and plant tolerance to low irrigation, making it valuable for agricultural and forestry purposes in the context of global warming.

## 1. Introduction

Cellulose, the major component of plant biomass and the most abundant biopolymer in the biosphere, is also produced by various organisms, including marine algae and prokaryotes [1]. In the nineteenth century, Adrian Brown identified bacterial-origin cellulose as a key component of the gelatinous membrane that forms on the fluid surface during vinegar fermentation produced by *Komagataeibacter xylinus* (formerly *Acetobacter xylinum* or *Gluconacetobacter xylinus*). *G. xylinus* is the most efficient cellulose-producing microorganism, which has become a model system for studying the biosynthetic mechanisms of BC in bacteria [2,3]. This study represented one of the earliest references to a bacterial biofilm. Notably, Brown detected cellulose in the pellicle, providing the first experimental evidence that implicates an exopolysaccharide in the formation of a bacterial multicellular community [4]. In nature, it is now well known to act as a molecular glue, facilitating biofilm formation and bacterial adherence [5,6].

Bacterial cellulose is also produced by the genera *Agrobacterium*, *Aerobacter*, *Rhizobium*, *Sarcina*, *Pseudomonas*, *Achromobacter*, *Alcaligenes*, and *Enterobacter* [7,8,9]. Unlike plant-origin cellulose (PC), BC does not require lignin and hemicelluloses and is synthesized as pure cellulose [10]. *G. xylinus* synthesizes a nanofibrillar film with a denser lateral surface and a gelatinous layer on the opposite side [11,12,13]. The process involves the polymerization of glucose residues into β-1-4 glucan, followed by extracellular secretion of linear chains, as well as the final crystallization of strips stabilized through hydrogen bonds and van der Waals forces [14]. 

In the past decade, BC has gained importance due to its extraordinary properties, including durability, biocompatibility, mechanical resistance, and moldability. In industry, BC has been used to restore paper structure [15] and to make eco-friendly textiles [16], nanomaterials [10], and nanocomposite films [17,18]. In biomedicine, BC is extensively used for wound dressings [19,20,21]. Recent Food and Drug Administration (FDA) approvals has expanded its use in the biomedical field [22]. By 2026, it is expected that the global market for cellulose could reach up to USD 305.08 billion (GVR—Grand View Research, 2016 [23]).

Although chemically identical to plant cellulose (PC), BC’s macroscopic and three-dimensional organization differs, resulting in a superior water retention than PC [24]. Because the hydrogel texture, BC holds up to 90 times its weight on water [25,26,27]. Its high biocompatibility and water retention make it suitable for cosmetics, including face masks and emulsion [28]. 

BC potential applications in agriculture are particularly noteworthy [29,30,31,32]. In soils, BC contributed to soil biodiversity being degraded by up to 75%, principally by fungi and Proteobacteria [33]. BC biofilms facilitate efficient plant–bacteria interaction, and depending on the host, BC producers exhibit a pathogenic or symbiotic relationship with plants. *Rhizobiaceae* colonize plant roots [34], while, for example, *Acetobacteriaceae* associate with insects and inhabit the carposphere [5].

For instance, BC has been shown direct antimicrobial properties against phytopathogens. Cellulose nanocrystals reduced, on leaves, the survival of *Pseudomonas savastanoi* pv. *savastanoi*, the causative agent of olive knot disease [35]. Additionally, BC silver nanoparticles, when applied as hybrid patches on plant foliage, enhance the efficacy of pesticides by reducing runoff. These nanoparticles have also demonstrated in vitro activity against significant phytopathogens such as *Botrytis cinerea* necrotrophic fungus and *Pseudomonas syringae* pv. tomato DC3000 biotrophic bacterium [36]. BC has also found applications on agriculture in food packaging, helping to reduce waste production [37,38]. Moreover, cellulose nanocrystals have been shown to be harmless to olive roots treated under hydroponic conditions, suggesting their potential use as future carriers for fertilizers or chemicals [5,39]. 

Despite its high potential applicability in agriculture and forestry, molecular studies on BC (bacterial cellulose) are primarily focused on bacterial metabolism, with limited research on the plant molecular response to BC. The phytohormone ethylene has been shown to enhance BC production in *Gluconacetobacter* by regulating the cellulose synthesis operon, while indole-3-acetic acid (IAA) downregulates it [40]. The authors hypothesize that during plant senescence, ethylene produced during ripening might contribute to bacterial chemotaxis and BC production for fruit colonization, providing an advantage against competing microorganisms in nature [5,40].

In this study, we present assays, conducted under controlled hydroponic conditions, demonstrating that pure BC is harmless to *Solanum lycopersicum* seedlings. Our results indicate that in soil, BC contributes to root growth and enhances plant tolerance to low-irrigation conditions. Additionally, BC increases the availability of key soil nutrients such as phosphorus (P), iron (Fe), and potassium (K). These findings underscore the potential of BC as a direct soil amendment to improve plant development and drought tolerance in the context of global warming.

## 2. Results

### 2.1. Bacterial Cellulose (BC) Structure, Composition, and Humidity Content

**Figure 1:**
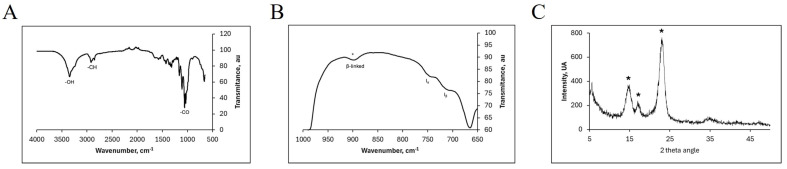
ATR-FTIR spectrum of bacterial cellulose (BC). (**A**) Whole spectrum. (**B**) Detail of the spectra between 650 and 1000 cm^−1^ showing two bacterial cellulose bands around 750 and around 710 cm^−1^, corresponding to the presence of the crystalline phases Iα and Iβ of BC, respectively. The presence of stars in FTIR spectra denotes the beta linkage of cellulose monomers. (**C**) XRD diffractogram recorded for the isolated bacterial cellulose, showing the crystalline structure of cellulose I with three main peaks hightlighted with stars located at 14.7, 17.3, and 23.2 corresponding to crystallographic planes 100, 010, and 110, respectively.

Since this study focuses specifically on the effects of bacterial cellulose (BC) on plants and substrates, it was crucial to obtain BC with the appropriate crystallinity. While plant cellulose is semicrystalline, bacterial cellulose exhibits high porosity and crystallinity, which significantly influences its biochemical properties [22,41]. The crystallinity of bacterial cellulose (BC) is crucial for maintaining its high water retention capacity. Plant cellulose fibers typically have diameters ranging from 13 to 22 µm and a crystallinity of approximately 44–65%. In contrast, bacterial cellulose fibrils have diameters of 10 to 100 nm, with a crystallinity of around 90%. Additionally, bacterial cellulose is highly hydrophilic due to the abundant hydroxyl groups on its surface [42,43]. Additionally, bacterial cellulose shows a higher level of purity and greater flexibility, when compared to cellulose from plant sources [43]. It has been described that both the water potential as well as the degree of hydration of plant and bacterial cellulose are affected by their composition [44]. A biochemical analysis was performed. Figure 1A shows the attenuated total reflectance-Fourier transform infrared (ATR-FTIR) spectrum of the BC. The characteristic bands of cellulose were observed, including skeletal vibrations involving C–O stretching around 1050 cm^−1^, C-H stretching of CH_2_ and CH_3_ groups around 2900 cm^−1^, and O-H stretching around 3340 cm^−1^. The bands around 1427, 1280, and 897 cm^−1^ are typical of the cellulose I allomorph. In Figure 1B, a detailed view of the spectra around 650 and 1000 cm^−1^ is presented, showing two bands around 750 and 710 cm^−1^, which correspond to the crystalline phases Iα and Iβ of BC, respectively [45]. 

Bands around 1536 and 1640 cm^−1^, identified with amide bonds due to the presence of proteins or residual biomass [46], were observed in our spectra. The low-intensity band around 1536 cm^−1^ indicates a high purity of the sample, reflecting a low presence of proteins, contaminants, or biomass. Figure 1C shows the X-ray diffraction (XRD) diffractogram recorded for the isolated bacterial cellulose, revealing the typical crystalline structure of cellulose I with three main peaks located at 14.7°, 17.3°, and 23.2°, corresponding to the crystallographic planes 100, 010, and 110, respectively [17,47]. These peaks confirm the presence of crystalline phases Iα and Iβ, consistent with the ATR-FTIR spectrum [48]. The crystallinity index was determined to be 84%, aligning with other reports for bacterial cellulose [15,39]. Notably, no residual salts from the growth media were detected, indicating the high purity of the isolated bacterial cellulose, making it suitable for later uses. Representative photos of BC obtained and analyzed during this process are shown in Figure S1.

The swelling capacity of the isolated bacterial cellulose was determined to be 3650 ± 412%, which is consistent with previously reported results showing the superabsorbent behavior of bacterial cellulose, particularly when samples are freeze-dried, as done in this work [39,49,50]. The bacterial cellulose showed a water uptake of 36.50 g of water/g polymer (standard deviation: 4.12).

### 2.2. Physiological Effects of Bacterial Cellulose on Solanum lycopersicum Seedlings

**Figure 2:**
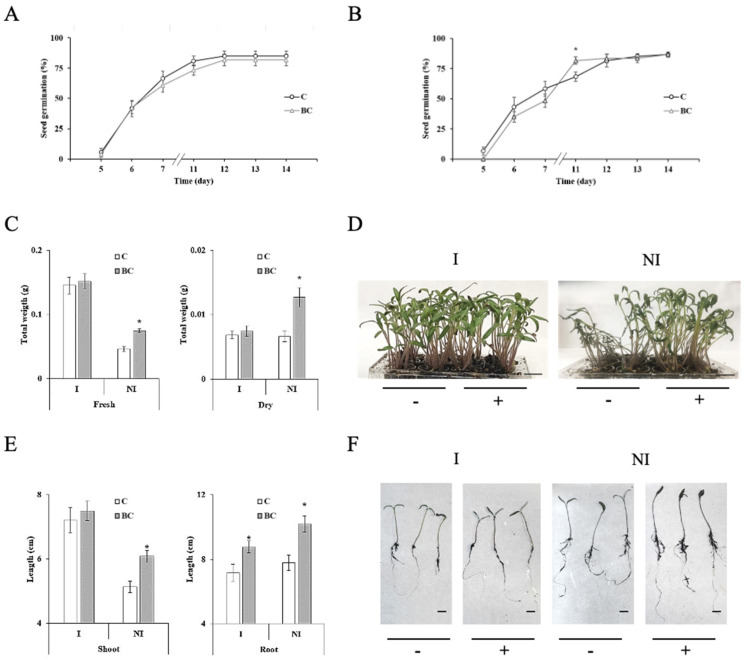
Physiological effects of bacterial cellulose (BC) on *Solanum lycopersicum* (Sl). (**A**) Seed germination rate on substrate containing 0.01% (*w*:*w*), BC (triangle), or watered control (circle). Data were collected up to 14 days. (**B**) Seed germination rate under hydroponic conditions in the presence of 0.01% (*w*:*v*), BC (triangle), or watered control (circle). Data were collected up to 14 days. (**C**) Total fresh and dry weight (g) of seedlings, growth under optimal irrigation conditions (I), or under regulated non-irrigation conditions (NI), in the absence (white) or presence of BC (grey). (**D**) Representative photos of 14-day-old seedlings growing at the same plate under I or NI conditions, in the absence (−) or presence (+) of BC. Bars: 1 cm. (**E**) Total shoot (left figure) and root (right figure) lengths (cm), measured in the absence (white) or presence of BC (grey) growth under I or NI conditions. (**F**) Detail of shoots and roots photos corresponding to (**E**), obtained for measuring seedling growth using ImageJ^®^ tool (1.53 version) (see Section 4) under I or NI conditions. Assays were performed at least three times with similar results using ten seedlings per pot and twelve pots per plate and tree plates per treatment (n = 120 per plate). Data were analyzed with the Stat-graphics Centurion 19 program, using a Variance check (*p* > 0.05) and a non-parametric Kruskal–Wallis test. * Significant statistical differences.

The physiological effects of BC on *Solanum lycopersicum* were studied based on biochemical properties. First, the effect of BC on seed germination was analyzed both on substrate containing 0.01% BC (see methodology), or under hydroponic conditions, treating the seeds to direct contact with 0.01% BC. The results shown in Figure 2A,B determined that the presence of BC does not produce seed death or improves germination, on both conditions, with no statistically significant differences related to controls in the absence of the polymer. However, significant differences observed only at eleven days are attributed to a delay on seed germination produced by BC, which recovered later, causing no significant differences on the global germination ratio. The germination was followed up to fourteen days [51]. Representative photos of media (substrate) in the absence and presence of BC and fourteen-day-old seedlings germinated on those media are shown in Figure S2.

Secondly, based on the high water retention capacity of the polymer, obtained in 36.50 g of water/g at this work, and on previous works under different conditions [27,52], we studied the physiological seedling response to BC, under optimal conditions of growth and irrigation (I), and under regulated stress non-irrigation conditions (NI) (see Section 4). As is shown in Figure 2C, BC produced any significant effect on total fresh or dry weight of seedlings, after fourteen days, under optimal conditions of water supply. However, a significant increase on both parameters was detected under non-irrigation (NI) conditions. Detailed photos of seedling phenotype growing on substrate in the presence (+) or absence (−) of BC (0.01%) are shown on Figure 2D, where it is shown that the survival of *Solanum lycopersicum* seedlings was improved after 14 days by a positive effect of BC under limited irrigation conditions (NI).

Additionally, an increase in root length was observed in the presence of bacterial cellulose (BC), with the effect being more pronounced under limited irrigation conditions (Figure 2E). Conversely, shoot length increased significantly only under non-irrigation stress conditions (Figure 2E). Detailed measurements of shoots and roots of seedlings under both conditions are shown in Figure 2F. The percentage changes in these parameters relative to control treatments are detailed in Table S1. To further confirm that BC enhances plant development, we analyzed the total carbon and nitrogen content in the seedlings. BC significantly increased carbon and nitrogen levels in plants under non-irrigation conditions (Figure S3).

### 2.3. Effects of Bacterial Cellulose into Organic Substrate 

Based on the biochemical characterization of bacterial cellulose (BC) conducted in this work, which highlights BC’s high water retention capacity, we investigated its effects on the substrate used for cultivating *Solanum lycopersicum*. While previous studies have shown that BC enhances the growth of soil bacterial and fungal communities [53], and that plant cellulose decomposition promotes microbial growth in tropical forest soils [54], there is limited information on the direct effects of BC on plants growing in substrates containing BC. For our experiments, we selected a professional-use organic substrate specifically designed for the cultivation of *Solanum lycopersicum* and other horticultural species. This substrate was supplemented with vermiculite to optimize humidity, germination, and growth (see Section 4).

**Figure 3:**
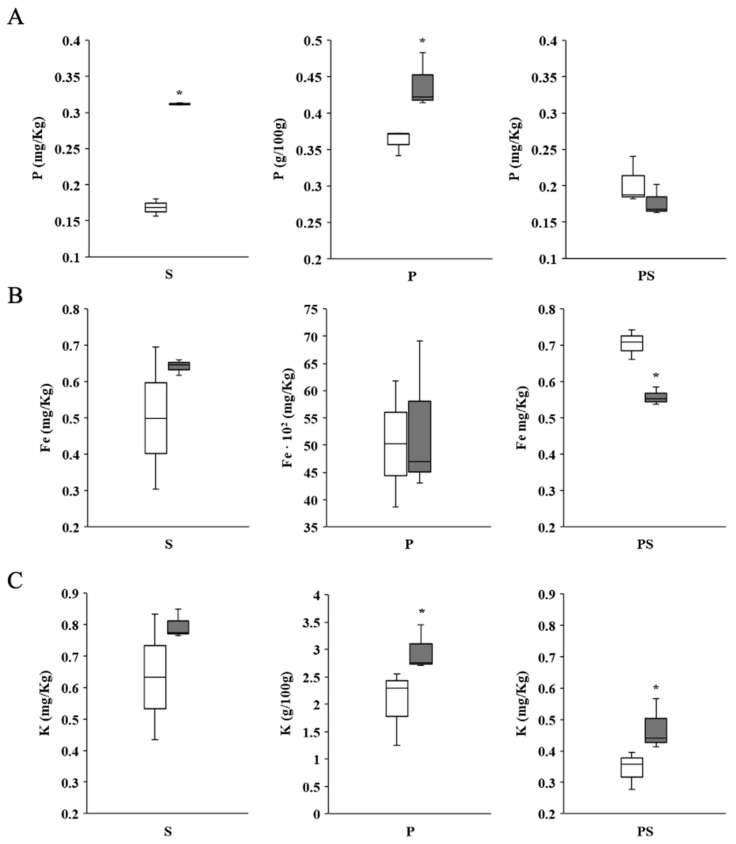
Effect of bacterial cellulose (BC) on Fe, K, and P content in plants and substrate. (**A**) Effect of BC (0.01%, *w*:*w*) on total P content on substrate (S, mg/kg), plants (P, g/100 g), or planted substrate (PS, mg/kg) in the absence (C) or presence of BC (0.01%, *w*:*w*). (**B**) Effect of BC on total Fe content on S (mg/kg), P (mg/Kg × 10^2^), or PS (mg/kg) in the absence (C) or presence of BC. (**C**) Effect of BC on total K content on S (mg/kg), P (g/100 g), or PS (mg/kg) in the absence (C) or presence of BC. Substrate and seedling samples were collected after fourteen days, making the treatment at time zero. At least three assays were performed analyzing ten seedlings per pot, three pots per treatment, with three plates (n = 120). Data were analyzed with the Stat-graphics Centurion 19 program, using a Variance check (*p* > 0.05) and a non-parametric Kruskal–Wallis’s test. White is control (C), while the grey bars correspond to bacterial cellulose (BC) treatment. * Significant statistical differences.

We analyzed the substrate (S) both with and without BC and examined the plant composition (P) after growth in the presence or absence of BC. Additionally, we evaluated the substrate where plants were grown (PS). Our findings, illustrated in Figure 3A, show that the inclusion of 0.01% BC in the substrate improved phosphate availability (P). This was reflected by higher phosphate levels in the BC-treated substrate (S) compared to the non-treated substrate. We also observed an increase in phosphate levels in the plants (P) and a reduction in phosphate levels in the planted substrate (PS).

A similar trend was seen for iron (Fe), although the increases in substrate and plant iron content were not statistically significant. However, a significant decrease in iron was noted in the substrate where plants were grown. Additionally, BC increased potassium (K) availability in both the substrate and the plants, with a notable rise in potassium levels in the planted substrate (PS). The percentage changes and significance of these parameters relative to control treatments are detailed in Table S2.

To evaluate how BC affects the water retention capacity of the substrate, we measured the water-holding capacity (WHC) with and without 0.01% BC (*w*:*w*). The results indicated that BC increased the WHC of the substrate by up to 14% compared to the non-treated substrate (Figure S4).

### 2.4. Molecular Response of Solanum lycopersicum to Bacterial Cellulose

While extensive molecular studies have characterized the mechanisms of bacterial cellulose (BC) synthesis, research on plant responses to BC remains limited [24,38,55]. This work aimed to characterize the effects of BC on *Solanum lycopersicum* seedlings under controlled hydroponic conditions and to elucidate the specific molecular changes induced by this biopolymer. 

**Figure 4:**
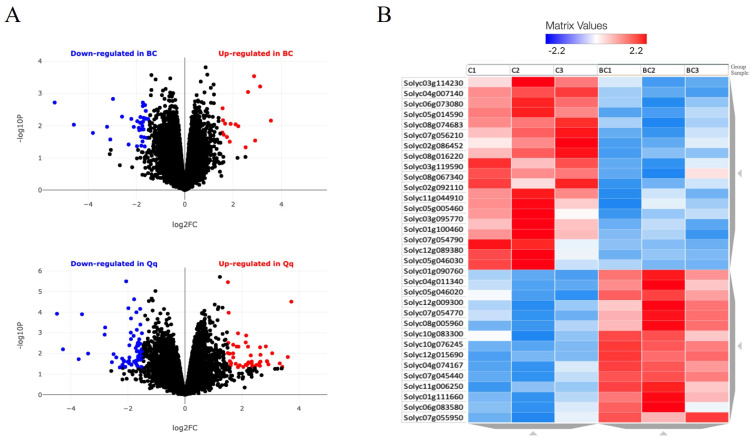
RNA sequencing data analysis of *Solanum lycopersicum* seedlings responding to bacterial cellulose (BC). (**A**) Volcano scatter plots obtained with BioJupies^®^ software, displaying the log2-fold changes calculated by performing a differential gene expression analysis (see Section 4). Red points indicate significantly up-regulated genes, while blue points indicate down-regulated genes. The upper plot shows results of seedlings responding to BC (0.01%, *w*:*v*) after one hour, related to watered non-treated seedlings. The lower plot shows a positive control for molecular plant response to chitin (Qq, 0.01%, *w*:*v*) (see Section 4). (**B**) Heatmap obtained with BioJupies^®^ software (see Section 4), displaying significant gene expression on selected genes in the RNA-seq dataset. Induced (red) or repressed genes (blue) (FDR< or >0.05) are shown after 1 h of treatment with BC. Every row of the heatmap represents a gene, and every column represents triplicates of controls (C1 to C3) and BC treatments (BC1 to BC3), correspondingly. Every cell displays normalized gene expression values. *Solanum lycopersicum* gene IDs are shown in the left column obtained by using the ITAG 3.2 genome annotation version. Black dots mean significant diferences on BC treated plants related to untreated plants lower than 1.5.

A volcano plot based on differentially expressed gene (DEG) analysis revealed that the number of genes significantly upregulated or downregulated by BC was lower than anticipated. Specifically, only 15 genes were induced, and 22 genes were repressed by more than 1.5-fold compared to controls, suggesting that BC has minimal impact on the plant at the molecular level. For comparison, a volcano plot generated after treatment with chitin polymer, a well-known microbe-associated molecular pattern used as a positive control, showed a greater number of upregulated and downregulated genes (Figure 4A).

KEGG pathway analysis (see Section 4) did not identify any significant metabolic pathways involved in the plant’s response to BC, nor did it reveal stress pathways within this limited set of genes. DEG analysis, based on the ITAG 3.2 version, is illustrated in the heatmap generated using BioJupies^®^ Software (version 4.0, https://maayanlab.cloud/biojupies/, accessed on 10 July 2024) (Figure 4B). However, an extended analysis that included genes induced or repressed by less than 1.1-fold revealed a broader response involving 2574 genes across various pathways. This analysis identified enrichment in Map kinase-mediated signaling pathways, including those related to ethylene and jasmonate, but not salicylic acid (Figure S6A). Additionally, genes associated with stress adaptation, cell enlargement, and plant growth (Figure S6B) and those involved in ubiquitin-mediated proteolysis in the proteasome (Figure S6C) were found to be enriched. 

## 3. Discussion

In this study, we synthesized bacterial cellulose (BC) in the laboratory, characterized its properties, and examined its effects on *Solanum lycopersicum* (tomato) seedlings, focusing on its impact on both the substrate and the plants themselves. Our analysis confirmed that the crystal structure of BC produced in our lab matched previously described structures [41], validating the effectiveness of our protocol. Additionally, BC exhibited a high water retention capacity of 36.50 g of water per gram of polymer, consistent with earlier studies [27,52] and surpassing the water retention capabilities of plant-derived cellulose [22,56]. 

Our findings revealed that BC significantly increased the water-holding capacity (WHC) of the substrate, which correlated with improved seedling survival under non-irrigation conditions. A previous study made on plant cell wall hydration made to study the direct effects of water deficit on the plant cell showed that the plant might respond to BC and PC, affecting their water-holding capacity, for example, producing expansins [44]. Despite this enhancement, no expansin-encoding genes were induced by BC, suggesting that expansins do not play a role in BC’s impact on WHC under our experimental conditions. BC appeared to be benign to the plants, with no stress symptoms observed in hydroponic or soil-based conditions. Although there was a slight delay in seed germination at seven days, this was not statistically significant, and germination rates normalized by eleven days. This delay may indicate that BC interacts with seed surfaces, warranting further investigation into potential receptors.

Our study aligns with previous findings that BC is non-toxic to plants, as similar results were observed with BC nanocrystals in olive roots [5,39]. Molecular analysis revealed that the most significantly repressed gene was pathogenesis related protein 1 (PR1), a known stress-related protein. PR1 is commonly induced by wounding, several stresses, and pathogens that activate the salicylic acid (SA)-mediated defense signaling pathway on plants [57] such as the well-studied phytopathogenic bacteria, *Pseudomonas syringe* pv tomato DC3000 [58,59]. This suggests that BC does not trigger typical stress responses associated with pathogens. 

Physiologically, BC positively influences root development under both optimal and limited water conditions, enhancing survival and root growth. This effect was notably pronounced under water stress, where BC facilitated increased root and shoot length and improved overall plant biomass. BC also improved nutrient availability, particularly phosphate, iron, and potassium, likely due to its water retention properties, which enhance root nutrient absorption. The addition of BC to the substrate increased WHC by up to 15%, compared to a 40% increase with vermiculite, indicating that BC is highly effective in enhancing WHC.

Further assays are needed to explore the specific interactions between BC and soil nutrients. Previous research has shown that BC could potentially chelate divalent cations, enhancing nutrient availability. Our findings suggest that BC’s effects on substrate and nutrient availability are multifaceted, involving complex interactions between substrate composition, biomass nature, and root interactions. 

In line with our results, previous work made with bacteria cellulose membrane produced by the bacterium *Acetobacter xylinum* showing up to 125% of water retention [52]. However, differences observed in water retention percentages depends on bacteria species and experimental conditions [22].

The analysis of the effect of bacterial cellulose (BC) on the substrate revealed that BC enhances nutrient availability for plants, specifically phosphate, iron, and potassium. This effect is attributed to the increased water retention facilitated by BC, which likely improves the root absorption of these nutrients. In this context, BC exhibits a dual effect by enhancing nutrient absorption through increased water-holding capacity (WHC) and promoting root growth. In our study, BC increased WHC by up to 15% compared to controls with only 0.01% (*w*:*w*) BC, while vermiculite alone increased WHC by up to 40% when used at 25% in the substrate. This indicates that the effect of BC is nearly one thousand times more efficient than that of vermiculite. In line with increasing in phosphate uptake, which is directly related to plant capacity to fix carbon and synthetize later carbohydrates, the total content on carbon and nitrogen was increased on seedlings growing in the presence of BC treated to controls, and this increase was significant under non-irrigation conditions, indicating that BC contributes to plant survival and photosynthesis under low water availability conditions.

Since phosphorus availability to plants is related to the solubility of metal phosphates in soil, additional assays are necessary to determine the specific effects of this natural polymer on nutrient interaction and water retention in soils. Previous studies have shown that *Bacillus subtilis* bacteria contribute to phosphorus availability in soil by enhancing biomass production [60]. Cellulose and lignin regulate the partitioning of phosphorus fractions by activating enzymatic bacterial activity. The solubility of inorganic phosphate on soil depends on microorganisms that produce organic and inorganic acids, making soluble inorganic phosphates available for plants; however, other organic acids in the soil act as chelating agents, accomplishing divalent cations that were in the soil as phosphate. This property of chelating divalent cations is a very well-known activity produced by glucose-formed biopolymers such as plant cellulose [61], chitin [62], and BC [63]. The presence of BC might chelate divalent cations of inorganic phosphate, making phosphate more available for plants. These findings suggest that the effects of BC on substrate and nutrient availability are very complex, involving several factors including the specific WHC of each substrate and composition, the nature of the biomass, and the root interaction with the polymer.

In this work, we also performed an RNA sequencing genome analysis of *S. lycopersicum* seedlings including shoot and roots, responding to BC, using Genome Analyzer Illumina patform (Illumina, Inc. San Diego, CA, USA), under hydroponic conditions in water, and at short times of one hour. Under these conditions, in which the seedlings are only exposed to BC, we determined that, surprisingly, BC likes to be practically innocuous for the plant. A few genes were induced more than three times, being related to the controls, and the initial KEGG analysis did not gave us relevant families. However, when we included the genes induced at least double related to controls, three signaling pathways showed enrichment according to the fact that BC activates on *Solanum lycpersicum* cell elongation and ethylene and jasmonate pathways, but not SA and ubiquitin-mediated protein degradation in the proteosome. Surprisingly, the transcriptional induction of genes related to those signaling pathways were related to plant development and not defense, indicating that the plant does not recognize BC as a component from phytopathogenic bacteria and more likely recognizes it as a symbiotic one. The enrichment in ubiquitination has not been described before. Further analysis is necessary to explain the molecular involvement of these pathways in plant responses to BC.

These results are according to the absence of molecular stress responses or stress symptoms on seedlings, observed alongside this work. The use of BC films protecting olive leaves against *Pseudomonas savastanoi* pv. *savastanoi* [35], as well as the use of patches reducing water runoff on leaves and increasing protection against phytopathogens in *Nicotiana benthamiana* and *Solanum lycopersicum* leaves [36], in the absence of plant damage by the polymer, are in line with the results obtained in this work, determining the absence of negative effects of BC on plants. Between the genes induced by BC confirmed by qRT-PCR, we must highlight the presence of the gene encoding for the transcription factor bHLH (basic/helix-loop-helix, *Solyc03g114230*); this gene was described as being induced in the presence of different concentrations of melatonin, which induced the increasing of plant growth plus PEG3000 artificial simulated drought [64]. This gene was involved in this work in terms of drought tolerance as well as in plant development, similarly with the physiological response observed in this work. The second gene to highlight is *SlDMR6-2* (*Solyc06g073080*), belonging to the family of 2-oxoglutarate oxidases (2OG)-Fe (II). The *Arabidopsis thaliana Downy mildew resistance 6* (*AtDMR6*) gene encodes a protein that acts as a negative regulator of defense response against *Powdery mildew* [65,66]. In Arabidopsis, the mutant *dmr6* was found to be resistant to *Downy mildew*, *Hyaloperonospora arabidopsidis*, *Phytophthora capsica*, and *Pseudomonas syringae*, associated with the salicylic acid inhibition signaling pathway [67,68]. In potato, CRISP/Cas9 mutation on this gene conferred blight resistance [69], and in tomato, the loss of function of a DMR6 ortholog conferred a broad spectrum of disease resistance [68]. However, in *Vitis vinifera* (Grapevine), two genes have been described as belonging to this family with very different functions [70]. While the VviDMR6-1 protein is involved in defense response, VviDMR6-2, which is the ortholog gene induced by BC in this work, is involved in plant development, having not been induced by *Downy mildew* [70]. In the present work, the transcriptional induction of *StDMR6-2*-like gene by BC seems to be more related to plant development and SA signaling pathway inhibition, which oppositely regulates auxin-mediated root growth [71,72]. This response is again correlated with the induction of ethylene and jasmonic acid mentioned, involved in cell elongation and root development. Previously [70], it was suggested that DMR6s proteins connect in some way the defense-related genes with development ones into a network. This hypothesis might be according to our results from studying *Solanum lycopersicum* molecular response to BC, wherein the DMR6 family might connect this network, although additional molecular analysis will be necessary to confirm our hypothesis. 

Our molecular analysis is in line with the hypothesis that *Solanum lycopersicum* might not recognize bacterial cellulose as a pathogenic, disease-associated molecular pattern (PAMP, DAMP), differentially from other very well-known PAMPs such as chitin polymer or bacterial protein flagellin, in which plants possess specific receptors [73,74,75,76]. In fact, under the same conditions, chitin was used as a positive control of molecular plant response not observed after BC treatment. However, BC might be recognized as a microbe-associated molecular pattern (MAMP) because in the transcriptomic analysis, some genes, specifically one of them, overlapped with Solanum lycopersicum responses to *Trichoderma atroviride*. Specifically, BC induced in plants a polyphenol oxidase precursor (PPO) involved in plant detoxification (*Solyc08g074683*), a gene involved in phenylpropanoid biosynthesis, which was described to be induced in tomato by beneficial *Trichoderma harzianum strain* T22 [77] and *Trichoderma atroviride* strain P1, which activates ethylene and jasmonate acid pathways but not the salicylic one [78]. This strain was isolated from wood chips and selected as an effective biological control agent against foliar and post-harvest pathogens, such as *B. cinerea*, and for use in cold storage [79]. This result might indicate that BC also might provide plant protection against plant cold or disease stress, similarly to what happens with Trichoderma but in the absence of microorganisms, as what happens with other microbe-associated molecular patterns (MAMPs) such as the well-known chitin [80]. It must be hihlighted that any stress was observed on plants along this study caused by BC. During the transcriptional study, a few genes were significantly induced or repressed under BC treatment, with a high variability between biological sample analysis, attributed to hydroponic culture conditions and variety used, with a higher number of genes induced by chitin as we expected, which was used in this study as a positive control of molecular plant response, a well-known elicitor on defense and development response on plants [81,82]. These results confirmed to us that the treatment made with BC was successful. One of the more surprising results found in the short list of induced genes was the *Solanum lycopersicum* orthologous gene to the *Arabidopsis thaliana* gene encoding for a phytosulfokine 4 precursor AtPSK4 (*AT3G49780*). This protein is involved in Arabidopsis in cell growth, having been a growth factor that overexpressed under a 35S promoter, developing the roots faster than the wildtype [83], as well as increasing cell wall development [84] and the maintenance of procambial cell identity [85]. These molecular results are according to the phenotype of plant response to BC observed in this work, showing higher development of roots and shoots, especially under water non-irrigation conditions. These results are in line also with previous assays performed with olive roots, under hydroponic culture, in contact with BC-based nanocrystals showing any stress responses [5].

Overall, our study demonstrates that BC positively affects plant growth and survival under water deficit conditions without causing stress or adverse effects. The results underscore BC’s potential as a biofertilizer and soil amendment in the context of climate change. Future research should focus on elucidating the molecular mechanisms of BC–plant interactions and its potential applications in sustainable agriculture.

## 4. Materials and Methods

### 4.1. Biological Material 

Untreated seeds of *Solanum lycopersicum* L., var. *marmande* were kindly provided by Ramiro Arnedo S.A (La Rioja, Spain). Seeds were stored and maintained at 4 °C until use. Bacteria *Komagataeibacter sucrofermentans* CECT-7291 were provided by Colección Española de Cultivos Tipo (CECT, Valencia, Spain).

### 4.2. Obtention of Bacterial Cellulose (BC) Stock

The bacteria were reactivated according to CECT instructions. The vial was opened in the cabin by thermal breakage. Subsequently, 1 mL of CECT10 medium (mannitol broth) was added, and the mixture was homogenized. A total of 100 uL of solution was used to inoculate mannitol agar plates, which were seeded with glass beads of 2.7 mm diameter (Carl Roth GmbH Co., Karlsruhe, Germany). After removing the glass beads, the plates were incubated in a Constant Temperature Shaking Incubator FS-70B (Huanghua Faithful Instrument Co., Ltd., Ripollet, Barcelona) at 30 °C for 4 days. A total of 3 mL of mannitol broth was added to the plates. We homogenized the colonies with the liquid. The liquid was collected and divided into safety microtubes. Aliquots of 200 uL were generated using 100 uL suspension and 100 uL 40% glycerol (Carl Roth GmbH Co., Karlsruhe, Germany). The microtubes were immersed in liquid nitrogen and stored at −80 °C. Bacterial cellulose (BC) was obtained following the previously described methodology [15,47,86,87,88,89]. Briefly, one 15 mL tube containing 5 mL of mannitol medium was inoculated with 1 aliquot of bacteria [90]. The tube was incubated at 30 °C for 13 days in a water bath Precisterm (JP Selecta SA, Barcelona, Spain) in the absence of light. One borosilicate bottle containing 100 mL of mannitol medium was inoculated with 1.33 mL of the 13 day culture. The bottle was covered with metal paper and incubated at 30 °C for 10 days in a water bath. The bacterial cellulose produced was collected with forceps and placed in a 50 mL tube. The tube was centrifuged in Mega Star 600R (VWR International LLC, Leuven, Germany) for 5 min at 4000 rpm at room temperature. The supernatant was removed. Then, 3 mL of 0.1 M NaOH (Panreac Química S.L.U., Barcelona, Spain) solution was added to the tube [91,92,93]. The tube was kept in a horizontal position and shaken in an orbital shaker Skyline S-3.02.20L (ELMI Ltd., Riga, Latvia) for 30 min at room temperature. The tube was centrifuged for 5 min at 4000 rpm at room temperature. The supernatant was removed. Following this, 3 mL of distilled water was added to the tube, and it was vortexed with Bio Vortex V1 (Boeckel & Co. GmbH & Co. KG, Hamburg, Germany) for 10 s, repeating the process 3 times. The tube was centrifuged for 5 min at 4000 rpm at room temperature. We then repeated the washing with water and centrifugation 3 times. After this, we removed the supernatant and kept the BC. The BC was placed in the freeze dryer (Telstar SA, Tarrasa, Spain) until complete dehydration (24 h). The dehydrated BC was mechanically broken with sterile zirconium beads dia 1.4–1.7 mm (Jyoti Ceramic Industries Pvt Ltd., Maharashtra, India). The small BC pellets obtained were stored in a closed tube at room temperature.

### 4.3. Plant Growth Conditions 

Seeds of *Solanum lycopersicum* (Ramiro Arnedo S.A., La Rioja) were soaked in type I distilled water and stratified by hydropriming for 24 h at 4 °C. A current professional-use organic substrate was selected onto the assays, designed to meet the cultivation requirements of the professional grower, following recommendations from the provider. This substrate is currently used in the greenhouses for growing *Solanum lycopersicum* seedlings, supplemented with vermiculite, contributing to optimal conditions of humify, germination, and growth. Briefly, the 3:1 soil was prepared by mixing 3 parts Professional Seed Pro5050 substrate composed by a multimix of N-P_2_O_5_-K_2_O (NPK 14-16-18, Projar S.A., Valencia) and 1 part vermiculite type 2 (particle size 0.5–3 mm, density 95–110 kg/m^3^, Projar S.A., Valencia), using 150 mL of 3:1 soil, mixed with 24 mL of sterile type I distilled water, to fill each 12-well plate. Ten seeds were placed per well, irrigated with 500 µL of sterile type I distilled water. A regular water supply started after 5 days, with a periodicity of 500 µL of sterile type I distilled water every 2 days per well. For the assays performed under stress-regulated non-irrigation conditions, the treatment consisted of stopping irrigation after seven days. Plates in all assays were placed into an Aralab^®^ digital-controlled plant growth chamber (Lisbon, Portugal) at 50% humidity (*v*/*v*), with a temperature of 24 °C during the day and 18 °C during the night, with a 16 h light/8 h dark photoperiod and light intensity of 150 μE·m^−2^ per second. 

### 4.4. Bacterial Cellulose Treatments 

Based on our previous experience working with glucose-based biopolymers, a concentration of 0.01% of BC was selected. This concentration was proved to be enough to induce both physiological [82] and molecular responses on model plants responding to the glucose-based biopolymer chitin under similar conditions of treatment used on previous works [80,82].

For determination of the direct effect of bacterial cellulose on seed germination, seeds were stratified at 4^0^C overnight and germinated in sterilized distilled water on paper filter discs in 12-well plates with ten seeds per well. The germination rate was measured on seeds in the presence or absence of 0.01% (p:v) of bacterial cellulose, using sterile type I distilled water as controls. A regular water supply was maintained with a periodicity of 500 µL of sterile type I distilled water every 2 days per well. For determination of the effect of bacterial cellulose on seed germination on the substrate, seeds were stratified before germination on the substrate in the absence or presence of BC as before, with constant water supply. In all assays, the seed germination was analyzed up to fourteen days when germination is done for this variety (CPVO_OCVV, 2021 [51]).

For molecular response analysis of the direct effect of BC on seedlings, fourteen-day-old seedlings were treated with 500 µL of 0.01% BC in a sterile type I distilled water. Control seedlings were treated similarly with same volume of water. As a positive control of plant molecular response activation, seedlings were treated similarly with 0.01% (*w*:*v*) of a chitin mix following our previous work [82]. Tissues were harvested after one hour in liquid nitrogen and saved at −80 °C until total RNA extraction. 

For determining of physiological effects of BC on *Solanum lycopersicum* seedlings and on the organic substrate, a mix of 0.01% (weight: weigh) of BC related to organic substrate was used. The 0.01% *w*/*v* BC concentration was applied to the (3:1) substrate and homogenized before watering with sterilized type I distilled water. These conditions allowed us to determine the effects of the biopolymer at very short times into a high number of plants, based on previous optimized protocols used on *Solanum lycopersicum* [94]. A total number of 120 seeds per plate and at least three plates per treatment with at least three independent assays were performed for each assay.

### 4.5. Physicochemical Characterization of Isolated Bacterial Cellulose 

Fourier transform infrared spectroscopy (ATR_FTIR) ATR-FTIR (Specac, Ltd., New England, UK) was used to identify functional groups of bacterial cellulose. Spectra were recorded between 4000 and 650 cm^−1^, averaging 32 scans with a resolution of 4 cm^−1^ with an Agilent Cary 630 FTIR spectrometer (Agilent, Santa Clara, CA, USA) [15]. The areas of absorbance bands around 710 cm^−1^ and 750 cm^−1^ were used to estimate the percentage of cellulose Iβ.

### 4.6. X-ray Diffraction (XRD)

XRD diffraction patterns were obtained using a Material Powder Diffractometer (X´Pert PRO MPD, Malvern PANanalytical Ltd., Malvern, UK) in a θ–θ configuration secondary monochromator with CuKα (λ = 0.154 nm) and a solid-state pixel detector, operating at 40 kV with a filament of 40 mA [95]. The diffraction data were collected from 2θ values 5° to 50°, where θ is the angle of incidence of the X-ray on the sample. The crystallinity index (CI) of isolated bacterial cellulose was determined by the following Equation (1):

(1) $$\mathrm{CI}\;(\%)=\frac{I200-Iam}{I200}\times 100$$

where I 200 is the maximum intensity of the (200) lattice diffraction at 2θ around 23° and I am is the intensity scattered by the amorphous part of the sample (the location of the amorphous material signal was considered at 2θ around 19°).

### 4.7. Bacterial Cellulose Humidity Content and Water Uptake

Sample humidity content was determined gravimetrically according to Equation (2).

(2) $$\mathrm{Humidity}\text{:}=\frac{W1-W2}{I200}\times 100$$

where w1 is the initial weight of the sample and w2 is the weight after the procedure. Briefly, samples were weighted and introduced in a vacuum oven at 105 °C for 5 h. After that, samples were allowed to cool down into a desiccator at room temperature, and the weight was measured again by keeping the samples in a closed container to avoid water uptake during weighting [17].

The weighted freeze-dried samples (Wdry) of the biopolymer were weighted (between 15–20 mg of polymer) and immersed in 15 mL of milli-Q water over 48 h until constant weight (Wwet) [39]. After filtering the media, the samples were weighted again. The water uptake was calculated according to the following Equation (3):

(3) $$\mathrm{Water}\;\mathrm{uptake}\;(\%)=\frac{Wwet-Wdry}{Wdry}\times 100$$

The bacterial cellulose showed a water uptake of 3650 g of water/g polymer (standard deviation: 4.12).

### 4.8. Substrate Water-Holding Capacity

Water-holding content was obtained following the previously described methodology [96]. Briefly, filter paper discs of 9 mm diameter were cut and weighed. Three types of samples were prepared: substrate (S); substrate-vermiculite (SV), (3:1, *v*:*v*); and substrate-vermiculite plus 0.01% BC (SVP, *w*:*w*). Saturation was performed by using water immersion in 30 mL of milli-Q water over 48 h until constant weight [39]. Two-gram quadruplicates of each type of sample were weighed. Each replicate was placed on a filter paper disc and placed in a glass funnel for saturation with 30 mL of distilled water type I. The saturated samples were weighed together with the filter paper and placed in an Aralab^®^ chamber as previously for three days. Every 24 h, the samples are weighed. After three days, the samples are dried overnight at 85 °C. The dry weight of each sample was taken.

Moisture content [97] was measured following Equation (4):

(4) $$Water\;Holding\;Content\;\left(WHC\right)\%=\frac{weight\;of\;moist\;soil\;\left(M\right)-weight\;of\;dry\;soil\;(D)}{weight\;of\;dry\;soil\;(D)}\cdot 100$$

For weight measurements, the soil wells were carefully removed, and the plants were carefully separated. The microtubes were labelled and weighed. Two plants were placed in each microtube, immediately closed before weighing. The microtubes were opened for drying at 85 °C for two days. To obtain the dry weight, the tubes were closed and weighed immediately.

### 4.9. Plant Fresh and Dry Weights

The fresh weight was measured using fourteen-day-old seedlings after different treatments. After a step for cleaning substrate residues from roots, samples were placed into previously weighted 1.5 mL labeled tubes before measuring the fresh weight. To measure the dry weight, fresh seedlings were subjected to oven-drying at a temperature of 85 °C for 2 days, before weighting the closed tubes, avoiding putative increasing of seedling weight by water uptake from air. Data were related to percentages, considering controls as 100%.

### 4.10. Nitrogen and Carbon Content Analysis 

The plant samples were subjected to oven drying at a temperature of 85 °C for a duration of two days. Following the drying process, the tissues were accurately weighed and combined to achieve a total of 1 g per sample and 4 samples for the treatment group. The dried tissues were then meticulously ground to a fine powder with particle sizes less than 150 µm using a porcelain mortar and liquid nitrogen. The concentrations of nitrogen and carbon were determined using a mass elemental analyzer for macro-samples (LECO CHN-600, Leco Corp. St. Joseph, MI, USA), according to the manufacturer’s instructions.

### 4.11. Availability of Elements on Substrate and Plants 

To determine available elements in soil, 2.5 g of soil was extracted with 25 mL of 0.05 M EDTA in centrifuge tubes. Tubes were shaken for an hour at 20 °C, centrifuged, and filtered through Whatman number 2 [98,99]. Elements were measured in the filtrates with an ICP-OES THERMO ICAP 6500 DUO (SpectraLab Scientific Inc., Markham, ON, Canada).

For the calculation of total element content on plants, seedlings were washed in deionized water, oven dried at 60 °C, weighed, and ground. All plant material was digested in concentrated HNO_3_ in a laboratory microwave system, and concentrations of various elements were measured with ICP-OES THERMO ICAP 6500 DUO [100,101,102].

### 4.12. Statistical Analysis

The Stat Graphics Centurion XVI.II program (Stat Point Technologies, Inc., Warrenton, VA, USA) was used for all data analysis related to plant growth and disease parameters. A one-way analysis of variance (ANOVA) and Duncan’s mean comparison test were performed for all experiments, as well as *t*-tests with a significance level of 0.05%. In the case of non-homogeneous variance, a nonparametric Kruskal–Wallis test was used.

### 4.13. RNA QRT-PCR Analyses

Quantitative Reverse Transcription-PCR analysis was performed for RNA-seq data validation. Total RNA was isolated from frozen fourteen-day-old seedling tissues including shoots and roots. TRIzol Reagent (Invitrogen^®^, Carlsbad, CA, USA) was used according to the manufacturer’s protocol along with chloroform. RNA samples were then treated with a High pure RNA isolation kit to remove trace amounts of genomic DNA (Roche, Manheimm, Germany). RNA samples were analyzed to check quantity using a nanodrop (UV–VIS ACTG Gene UVS—99. 200 to 850 nm), and quality was checked using Qubit 4.0 (Fisher Scientific, Madrid, Spain). RNA samples were visualized in 1% agarose gel before next step staining with GelRed (Nippon, Japan). First-strand cDNA synthesis was primed using a hexanucleotide random primer, and cDNA was synthesized using a First-Strand Synthesis Kit (Amersham-Pharmacia, Rainham, UK), according to the manufacturer’s protocol. A 1.5 μL aliquot of the first-strand synthesis reaction was used as the template for PCR amplification. The program consisted of 3 min at 95 °C and 40 cycles of 30 s at 95 °C, 30 s at 60 °C, with a final extension step consisting of 7 min at 72 °C and dissociation melting curves. The quantitative real-time (qRT-PCR) experiments were performed using a SYBR^®^ Green qPCR master mix (Nzytech, Lisbon, Portugal) with reactions at a final volume of 10 μL per well. Samples were run in a DNA Engine One-Step QRT-PCR machine (Thermofisher Scientific, Waltham, MA, USA). Gene-specific primers were designed using the Primer Express 2.0 program (Applied Biosystems, Foster City, CA, USA), and minimal self-hybridization and dimer formation of primers were determined using the Oligo 6.0 program (Molecular Biology Insights, West Cascade, CO, USA). Primers with annealing temperatures of 58–60 °C that amplified products with lengths of about 150 bp were selected and then verified for specificity using a Basic Local Alignment Search Tool (BLAST+ 2.15.0) (https://blast.ncbi.nlm.nih.gov/Blast.cgi, accessed on 10 July 2024). The amplification efficiency for each pair of oligonucleotides was calculated as recommended by the manufacturer (Bio-Rad, Hercules, CA, USA), selecting only oligonucleotides with efficiencies above 90% for assays [103,104,105]. 

Gene specific primers used for quantitative real-time PCRs cited within the article are detailed in Table S3. Data were acquired using the One-Step PCR Applied Biosystem Analysis software (Version 2.01), and changes in transcript levels were determined using the 2^−∆∆CT^ method [106]. Data points were compared using *t*-tests. Three independent biological replicates from different assays were used with three technical replicates in each experiment. A regression line was calculated to analyze the correlation between Log_2_ RNA-seq readings and quantitative real-time PCR Ct results from twelve independent RNA samples and five genes for each tissue (Figure S4). Three independent biological replicates were analyzed for each sample. 

### 4.14. Construction of RNA-Seq Libraries

Total RNA from three independent biological replicates was extracted as detailed previously. For each sample, 1 μg of total RNA was used to construct the Illumina sequencing libraries according to the manufacturer’s instructions (TruSeq Stranded mRNA LT Sample Prep Kit, Illumina, Inc. San Diego, CA, USA). Libraries were sequenced using the Illumina HiSeq 2500 platform (Biomarker Technologies, Rohnert Park, CA, USA), and 150 bp paired-end reads were generated. 

### 4.15. Analysis of RNA-Seq Data

About 4 Gb of high-quality 150-bp paired-end reads were generated from each library, and the quality of the clean reads was checked using the Q < 20 threshold. To reduce analysis bias, artifacts such as low-quality reads, adaptor sequence, contaminant DNA, and PCR duplicates were removed using Cutadapt (https://pypi.org/project/cutadapt/, accessed on 10 July 2024). Trimmed reads were mapped to the reference genome with HISAT2 splice-aware aligner [107]. The *Solanum lycopersicum* reference genome and gene model annotation files were downloaded from the genome website browser (SGN release version SL2.50, https://solgenomics.net, accessed on 10 July 2024)). Known genes and transcripts were assembled using String Tie with aligned reads [108] based on the reference genome model (SL2.50). After assembly, gene/transcript abundance was calculated in the read count and normalized values were obtained, i.e., FPKM (fragments per kilobase of transcript per million mapped reads) and TPM (transcripts per kilobase million) for each sample using the feature counts function of the Bioconductor [109] package R subread [110] (strand Specific = 0, is Paired End = TRUE, require Both Ends Mapped = TRUE, primary Only = TRUE, ignore Dup = TRUE). Differentially expressed genes (DEGs) between samples were identified using the DESeq2 package [111] with standard parameters (fold-change was ≥ 1 and FDR-adjusted *p* value < 0.05). Average gene expressions in the three biological replicates were used for DEG identification using the *Solanum lycopersicum* ITAG 3.2 genome version publicly available.

### 4.16. Gene Ontology (GO) Enrichment Analysis and KEGG Pathway Analysis

Panther GO (http://www.pantherdb.org, accessed on 10 July 2024) was used for Gene Ontology (GO) enrichment. In this analysis, the GO enrichment analysis provided any GO terms significantly enriched in the DEGs relative to the genomic background. DEGs were filtered according to cellular components, molecular functions, and biological processes using the *Solanum lycopersicum* ITAG 3.2 genome version that is publicly available. KEGG (Kyoto Encyclopedia of Genes and Genomes; http://www.genome.jp/kegg/, accessed on 10 July 2024) is a main pathway-related database.

## 5. Conclusions

In this study, we successfully produced bacterial cellulose (BC) in the laboratory and analyzed its physiological and molecular effects on *Solanum lycopersicum* seedlings. We also examined the impact of the polymer on a current substrate. BC was found to contribute positively to root growth without inducing any molecular stress responses in plants, proving to be innocuous. Additionally, BC enhanced nutrient availability in the substrate, increasing the phosphate, potassium, and iron content in plants. Furthermore, BC improved plant tolerance to low-irrigation conditions. Our findings demonstrate and confirm that BC is a biopolymer with significant potential in agriculture and forestry to address the challenges posed by climate change.

## Data Availability

The data presented in this study are available upon request from the corresponding author due to privacy restrictions. Data supporting RNA sequencing analysis belong to Arquimea Agrotech^®^ S.L.

## Supplementary Materials

The following supporting information can be downloaded at https://www.mdpi.com/article/10.3390/plants13152158/s1. Figure S1: Detail of bacterial cellulose (BC) obtention. Figure S2. Microplate media and growth of *Solanum lycopersicum* seedlings. Figure S3. Effect of bacterial cellulose (BC) on total nitrogen and carbon content of *Solanum lycopersicum*. Figure S4. Water-holding capacity (WHC) of the substrate (S) in the absence or presence of bacterial cellulose (BC). Figure S5. Validation of RNA sequencing data by qRT-PCR. Figure S6A. MAPK signaling pathway enrichment analyses. Figure S6B. Plant hormone signal transduction pathway enrichment analyses. Figure S6C. Protein processing in endoplasmic reticulum pathway enrichment analyses. Table S1. Physiological effects of bacterial cellulose on fourteen-day-old *Solanum lycopersicum* seedling growth under irrigation (I) or regulated non-irrigation (NI) conditions. Table S2. Effect of bacterial cellulose on Fe, K, and P content in soil (S), plants (P), and planted soil (PS). Raw data (upper panel) and percentages (%) related to controls (lower panel) are shown. Table S3. qRT-PCR primers used in this study. References [94,103,104,105,112,113] are cited in the Supplementary Materials.
