# Supplementary figures and images for: Enhancing Solanum lycopersicum Resilience: Bacterial Cellulose Alleviates Low Irrigation Stress and Boosts Nutrient Uptake
Source: Plants (Basel). 2024 Aug 4;13(15):2158. doi: 10.3390/plants13152158 (PMC11313925; doi:10.3390/plants13152158)

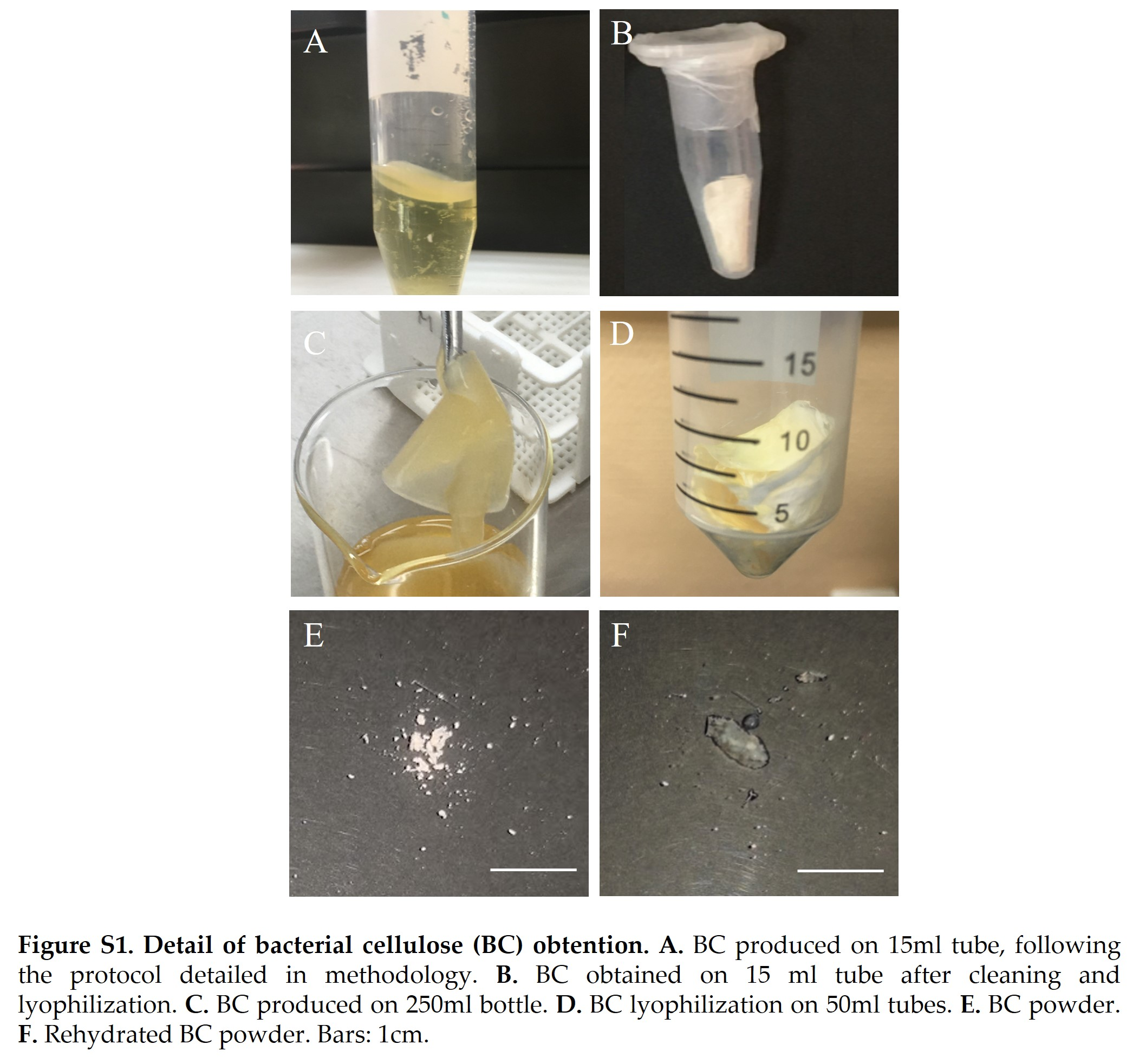

Supplement: Supplementary file 1 [file plants-13-02158-s001.zip › FigureS1.tif]

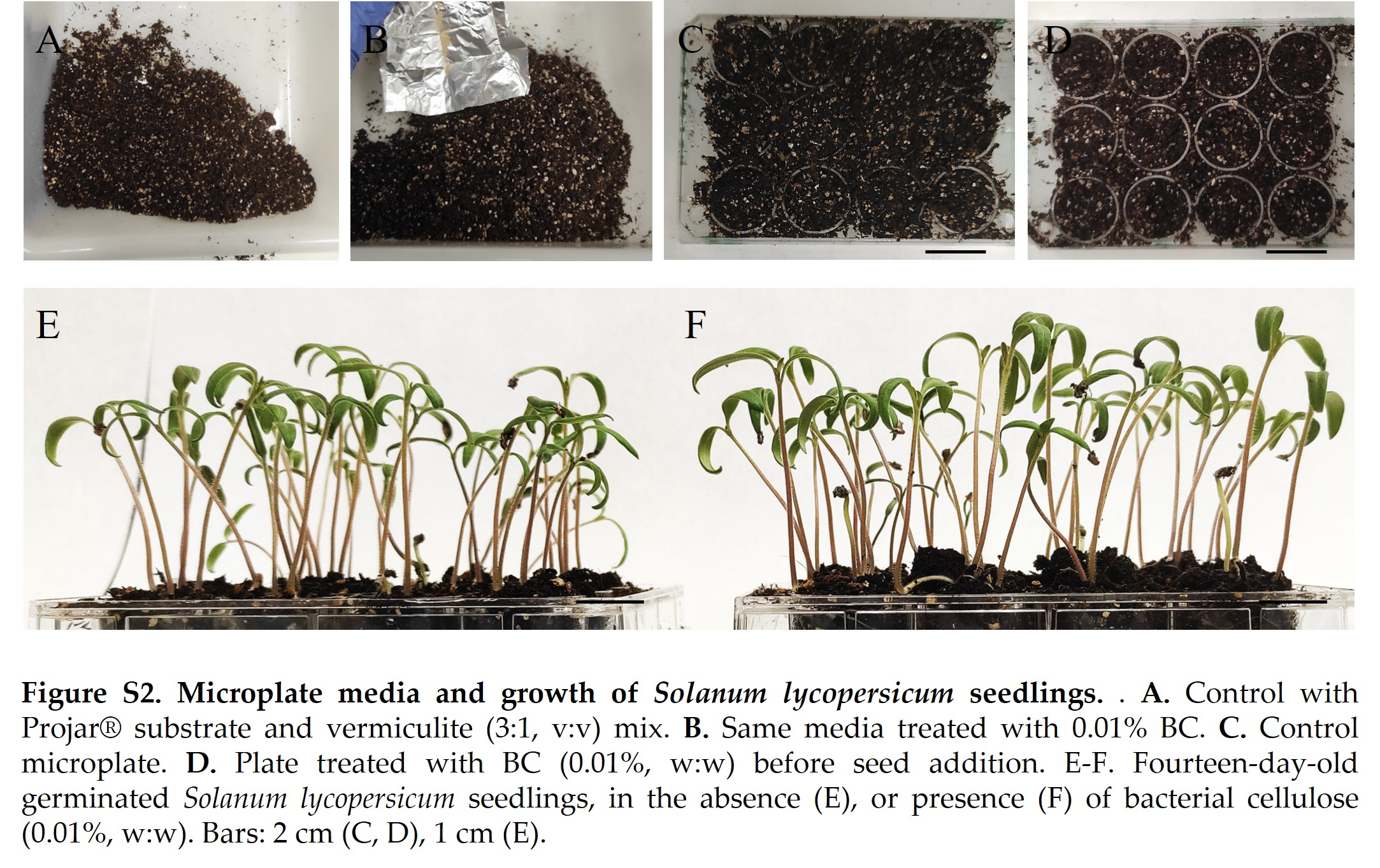

Supplement: Supplementary file 1 [file plants-13-02158-s001.zip › FigureS2.tif]

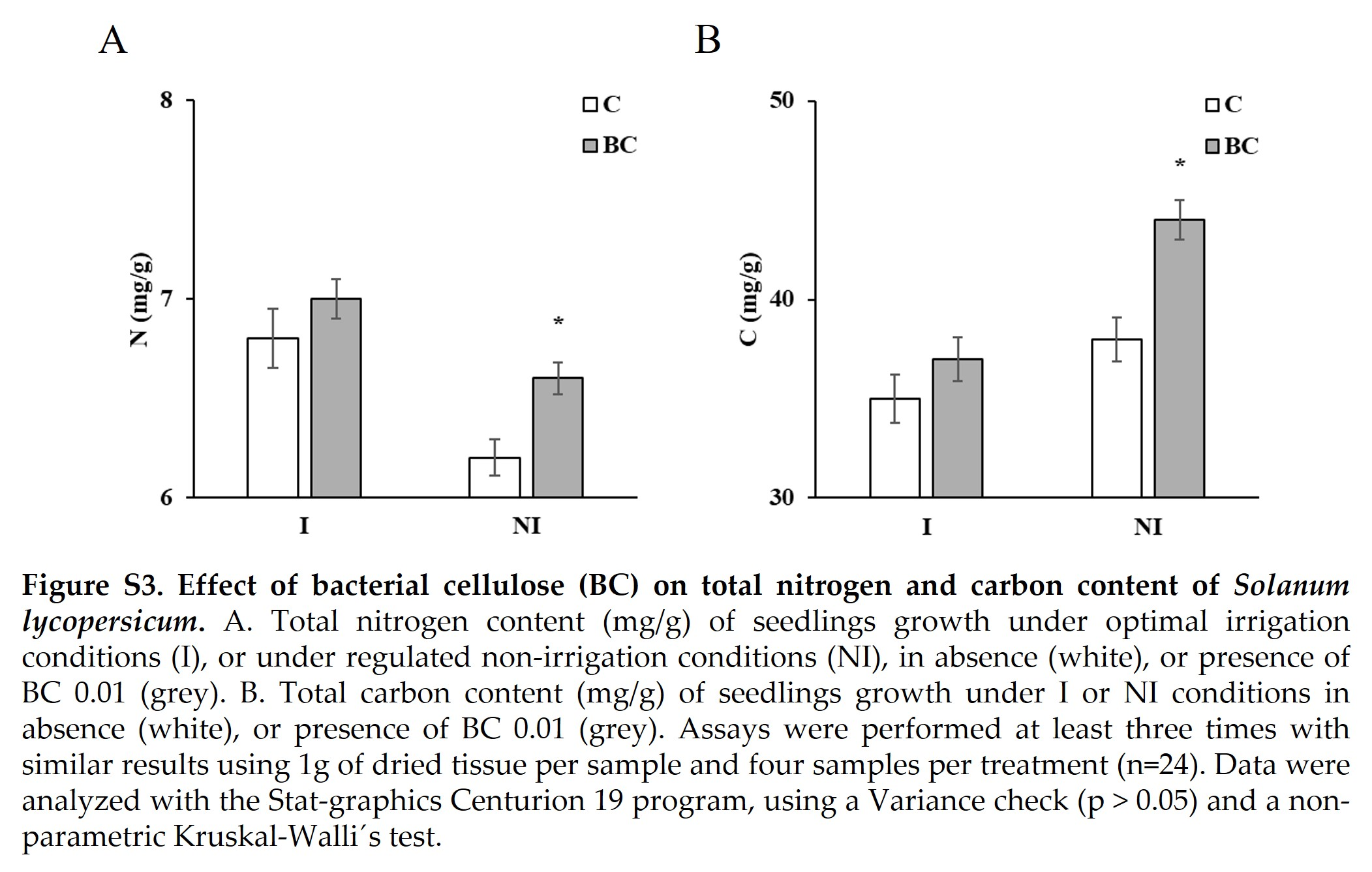

Supplement: Supplementary file 1 [file plants-13-02158-s001.zip › FigureS3.tif]

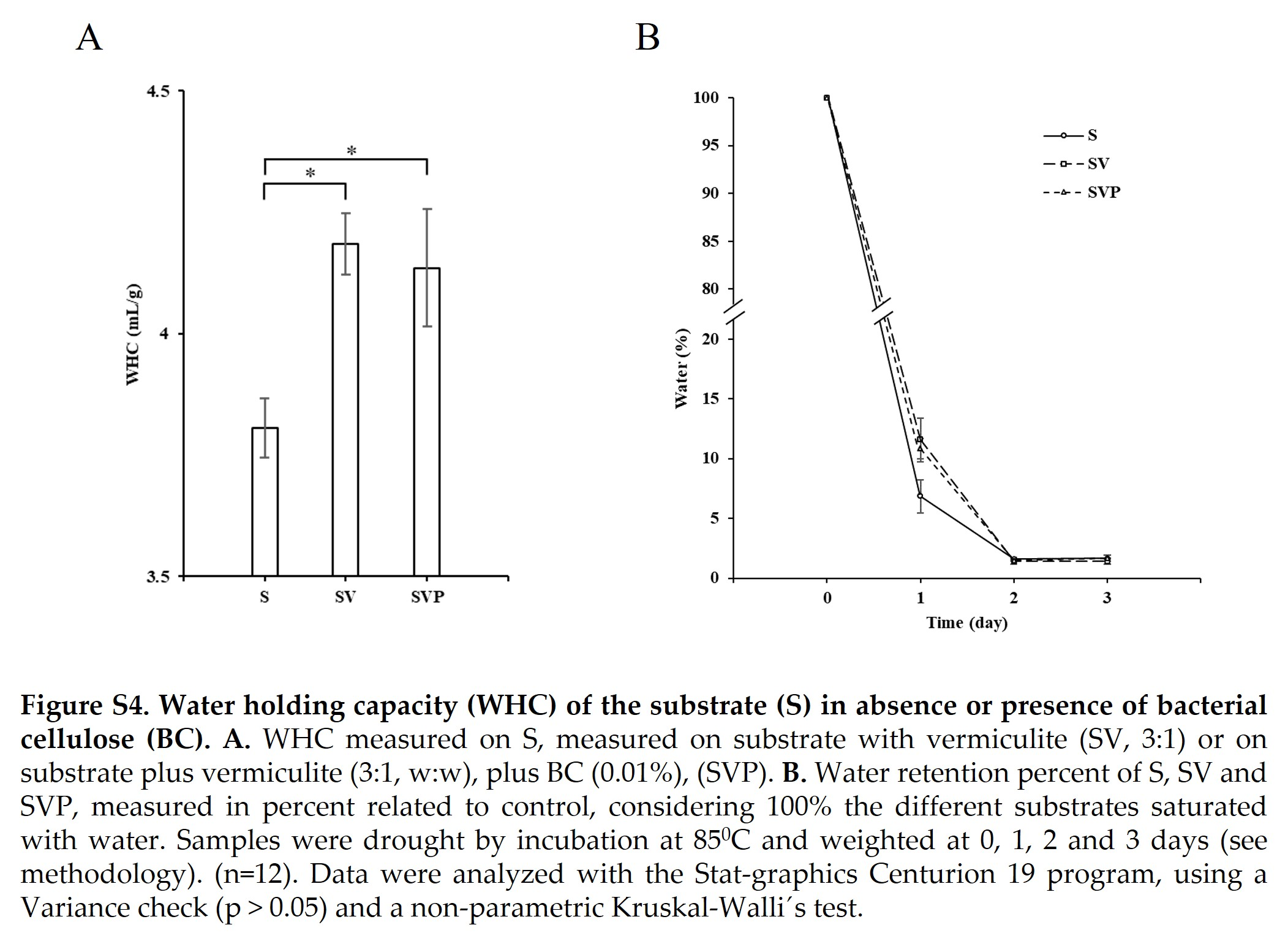

Supplement: Supplementary file 1 [file plants-13-02158-s001.zip › FigureS4.tif]

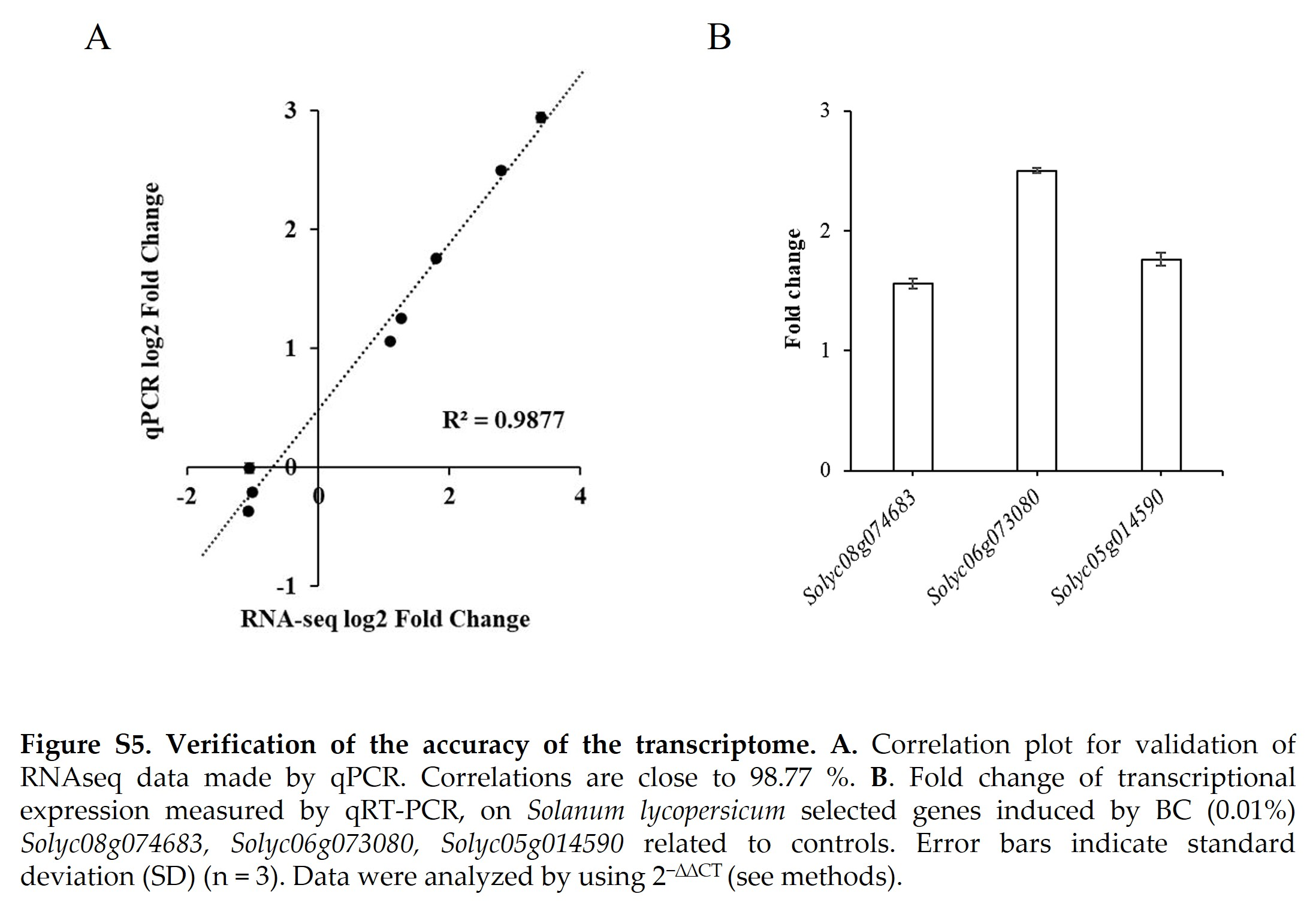

Supplement: Supplementary file 1 [file plants-13-02158-s001.zip › FigureS5.tif]

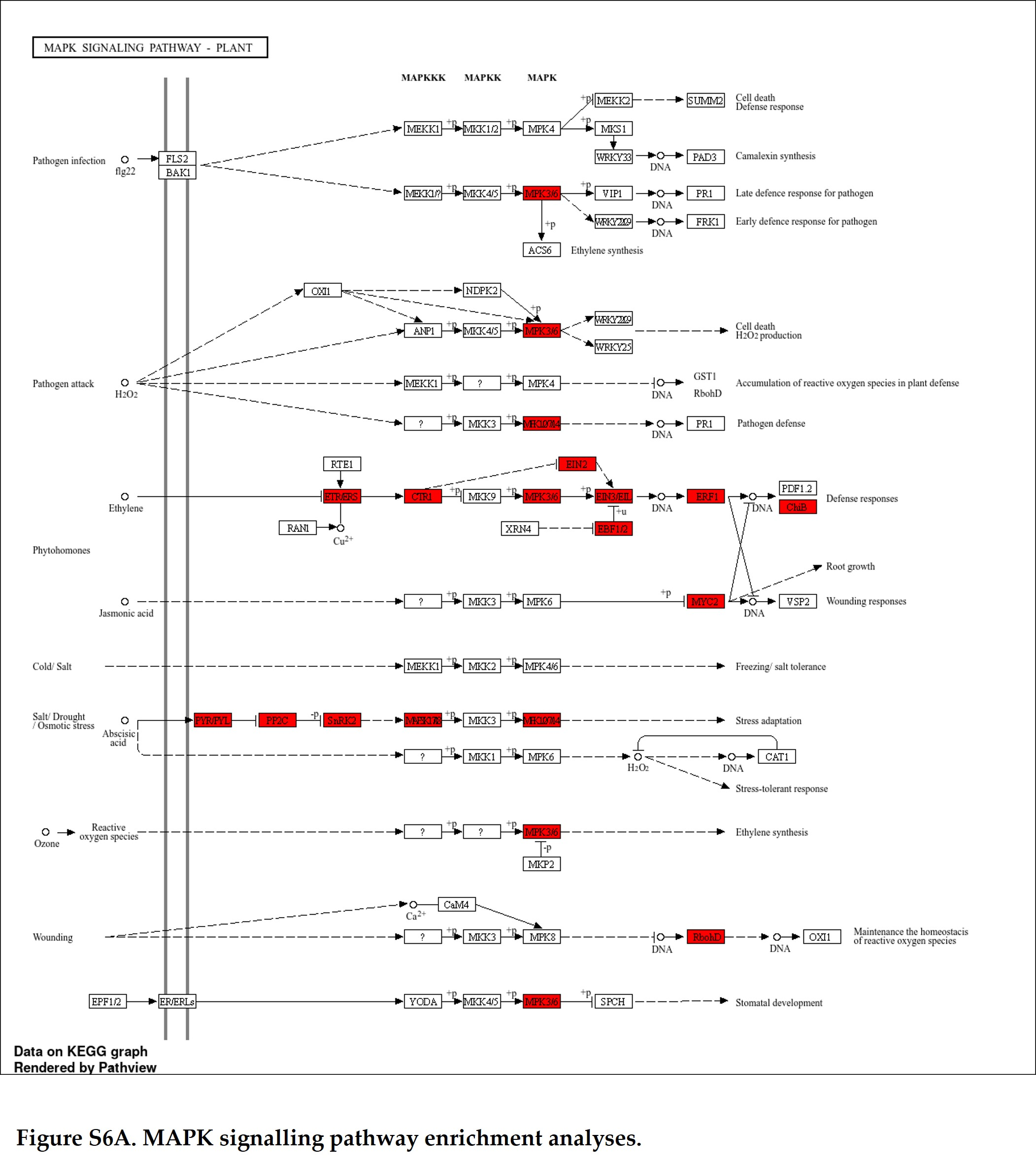

Supplement: Supplementary file 1 [file plants-13-02158-s001.zip › FigureS6A.tif]

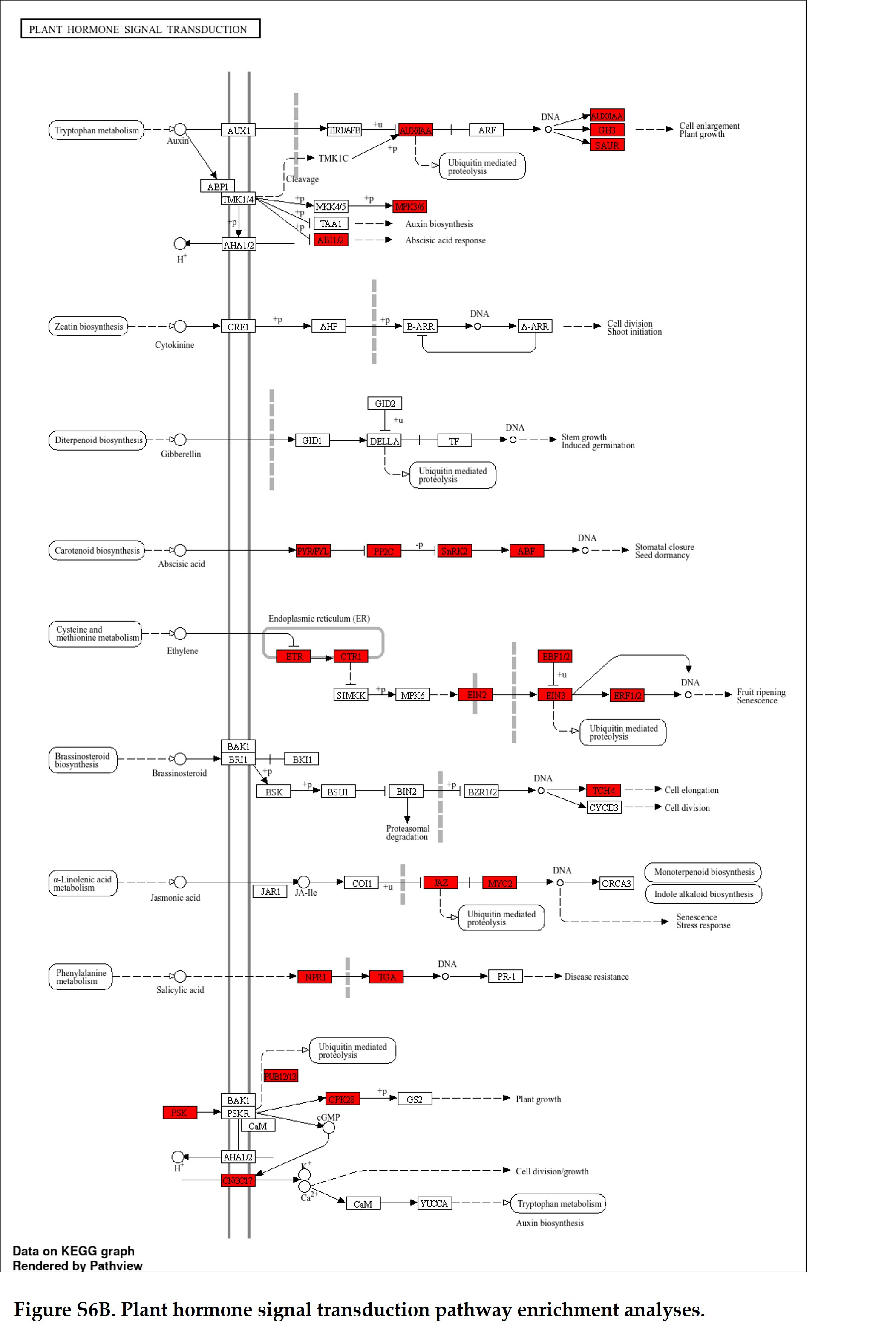

Supplement: Supplementary file 1 [file plants-13-02158-s001.zip › FigureS6B.tif]

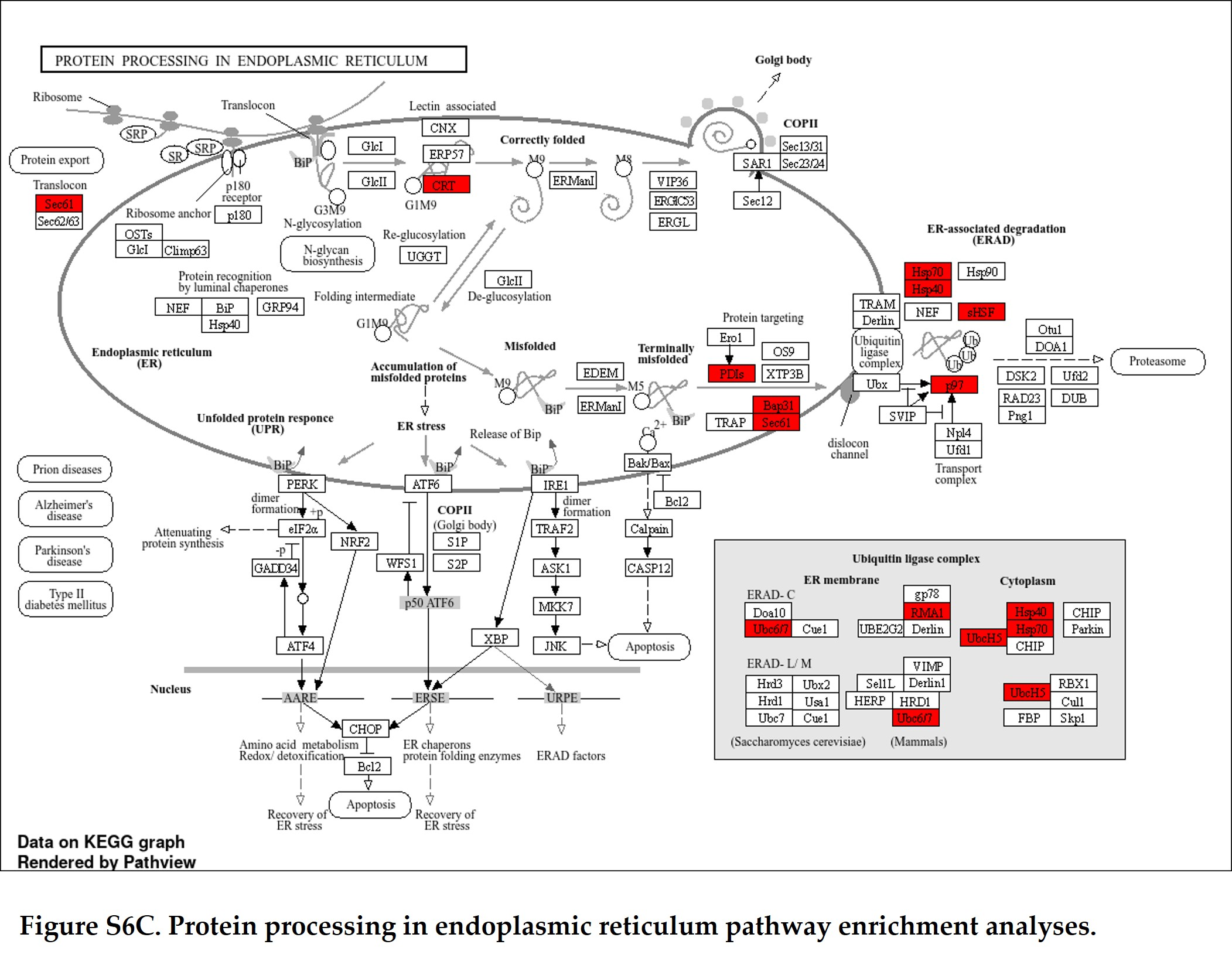

Supplement: Supplementary file 1 [file plants-13-02158-s001.zip › FigureS6C.tif]

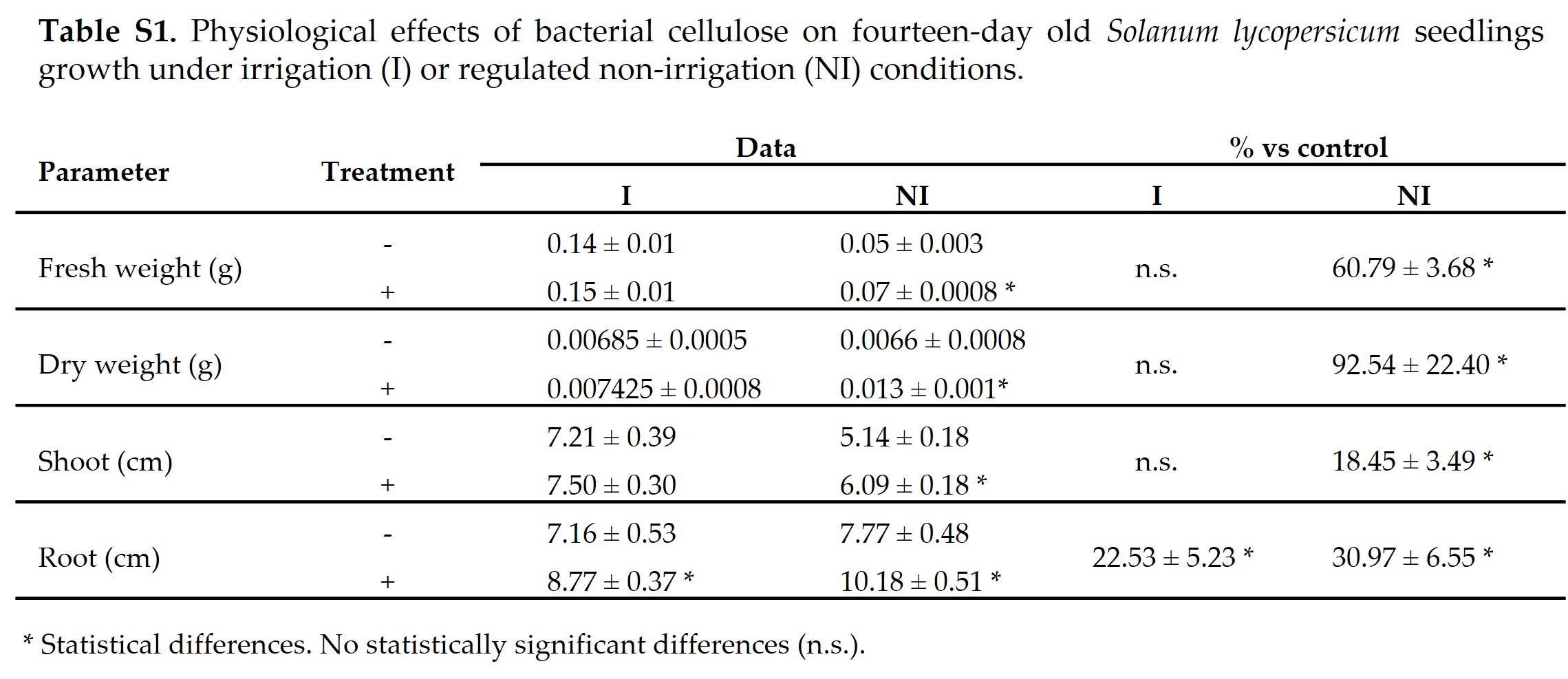

Supplement: Supplementary file 1 [file plants-13-02158-s001.zip › TableS1.tif]

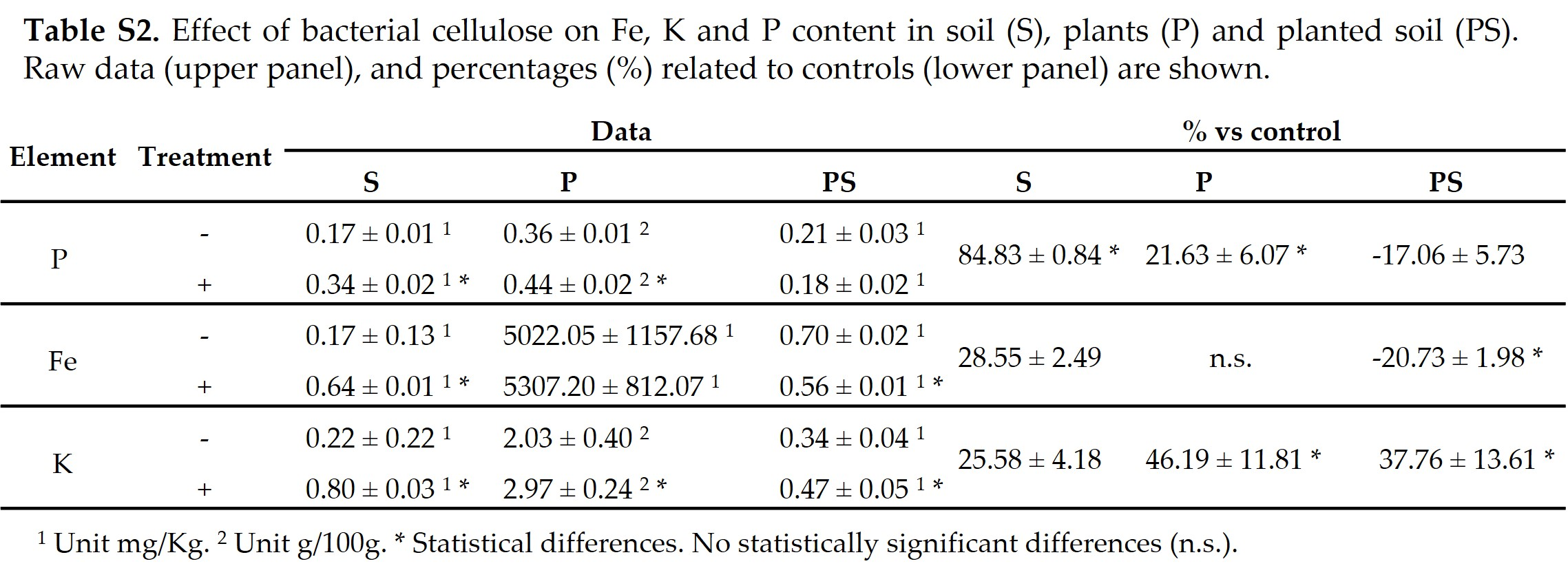

Supplement: Supplementary file 1 [file plants-13-02158-s001.zip › TableS2.tif]

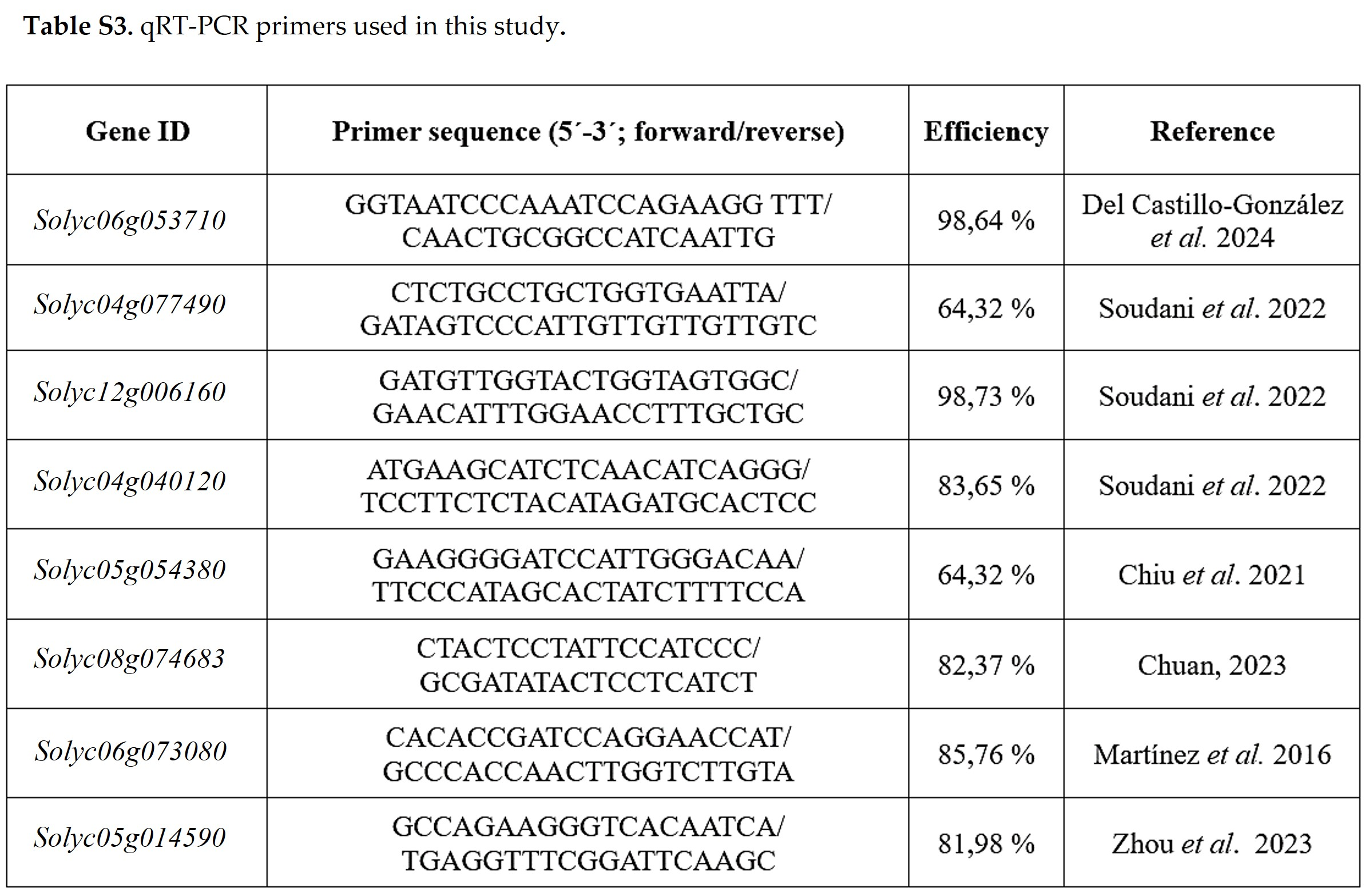

Supplement: Supplementary file 1 [file plants-13-02158-s001.zip › TableS3.tif]
